# Educational Session at the “Trends in Medical Mycology” (TIMM) 2021 Congress Teaching Medical Mycology to Students of Medicine

**DOI:** 10.3390/jof7110953

**Published:** 2021-11-10

**Authors:** Esther Segal

**Affiliations:** Department of Clinical Microbiology & Immunology, Sackler School of Medicine, Tel Aviv University, Tel Aviv 6997801, Israel; segale@tauex.tau.ac.il

Medical Mycology is part of Medical Microbiology. Medical Microbiology is considered a prerequisite of the curriculum for students of Medicine at the preclinical study stage. This stage of education of students in Medicine will form the basis for education on Infectious Diseases, a subject that will be taught at the clinical studies stage, including clerkships at rounds at the patient’s bed.

This basic dogma may result in different approaches to teaching Medical Mycology, such as detailed exhaustive studies during the initial preclinical study, or minimal studies at this stage and more in-depth and broader content during the clinical-study period.

The Educational Session at the TIMM 2021 Congress focus on the Teaching of Medical Mycology at the preclinical study stage, as exemplified by the author’s longstanding experience of teaching within the frame of a Medical School.

Teaching Medical Mycology, presented herewith, is composed of lectures and practical laboratory sessions, visualizing the subjects taught in lectures. Lectures include slide or video presentations, or other current media used in teaching methodologies. 

The presented curriculum includes the following topics:

1. The Introductory part of the course entails a discussion of the morphological and physiological characteristics of fungi, fungal ecology and geographic distribution, as well as the virulence-attributes of the fungi involved in human-diseases. The host–pathogen interactions and host’s immune response are also discussed.

This part also includes the diagnostic principles of fungal infections as well as the basic information on the current antifungal therapy of mycoses, discussing the major antifungal groups in use.

2. Lectures also include: the epidemiological aspects of host-pathogen interactions; the laboratory diagnosis, including laboratory test demonstrations, and the principles of treatment.
3. The major groups of fungi associated with human diseases and the mycoses caused by these fungi are covered.

These include: the dermatophytes and dermatophytoses, mallassezia infections, *Candida* and candidiasis, *Cryptococcus* and cryptococcosis, *Aspergillus* and aspergillosis, *Mucorales* and mucormycosis, the dimorphic fungi—emphasis on histoplasmosis and coccididomycosis, pneumocystis.

4. Each group of fungi is illustrated by a specific clinical-case description before the detailed characteristics are discussed.
5. Lectures are followed by laboratory sessions to demonstrate the characteristics of the major fungal pathogens.

We believe that this approach provides a sound basis of knowledge and understanding to the students for their later education at the clinical study stage, which follows the preclinical study period. 

